# 2-*O*-*β*-d-Glucopyranosyl-4,6-dihydroxybenzaldehyde Isolated from *Morus alba* (Mulberry) Fruits Suppresses Damage by Regulating Oxidative and Inflammatory Responses in TNF-α-Induced Human Dermal Fibroblasts

**DOI:** 10.3390/ijms232314802

**Published:** 2022-11-26

**Authors:** Kang Sub Kim, Yea Jung Choi, Dae Sik Jang, Sullim Lee

**Affiliations:** 1 College of Korean Medicine, Gachon University, Seongnam 13120, Republic of Korea; 2 Department of Biomedical and Pharmaceutical Sciences, Graduate School, Kyung Hee University, Seoul 02447, Republic of Korea; 3 Department of Life Science, College of Bio-Nano Technology, Gachon University, Seongnam 13120, Republic of Korea

**Keywords:** *Morus alba*, phenolic aldehyde, human dermal fibroblasts, tumor necrosis factor-α, skin damage

## Abstract

Human skin is composed of three layers, of which the dermis is composed of an extracellular matrix (ECM) comprising collagen, elastin, and other proteins. These proteins are reduced due to skin aging caused by intrinsic and extrinsic factors. Among various internal and external factors related to aging, ultraviolet (UV) radiation is the main cause of photoaging of the skin. UV radiation stimulates DNA damage, reactive oxygen species (ROS) generation, and pro-inflammatory cytokine production such as tumor necrosis factor-alpha (TNF-α), and promotes ECM degradation. Stimulation with ROS and TNF-α upregulates mitogen-activated protein kinases (MAPKs), nuclear factor kappa B (NF-κB), and activator protein 1 (AP-1) transcription factors that induce the expression of the collagenase matrix metalloproteinase-1 (MMP-1). Moreover, TNF-α induces intracellular ROS production and several molecular pathways. Skin aging progresses through various processes and can be prevented through ROS generation and TNF-α inhibition. In our previous study, 2-*O*-*β*-d-glucopyranosyl-4,6-dihydroxybenzaldehyde (GDHBA) was isolated from the *Morus alba* (mulberry) fruits and its inhibitory effect on MMP-1 secretion was revealed. In this study, we focused on the effect of GDHBA on TNF-α-induced human dermal fibroblasts (HDFs). GDHBA (50 μM) inhibited ROS generation (18.8%) and decreased NO (58.4%) and PGE_2_ levels (53.8%), significantly. Moreover, it decreased MMP-1 secretion (55.3%) and increased pro-collagen type I secretion (207.7%). GDHBA (50 μM) decreased the expression of different MAPKs as per western blotting; p-38: 35.9%; ERK: 47.9%; JNK: 49.5%; c-Jun: 32.1%; NF-κB: 55.9%; and cyclooxygenase-2 (COX-2): 31%. This study elucidated a novel role of GDHBA in protecting against skin inflammation and damage through external stimuli, such as UV radiation.

## 1. Introduction

The skin is the outermost organ and makes up approximately 10% of the human body. It is divided into three layers: epidermal, dermal, and subcutaneous tissues [1]. The dermis is composed of the ECM (extracellular matrix), which is composed of proteins, such as collagen and elastin [2], with each component having a distinct function. Collagen supports the skin and makes up 70–80% of the dry weight of the skin; elastin provides elasticity and flexibility in the skin and makes up 2–4% of the ECM. These functional effects are dependent on various groups of dermal cells, such as dermal fibroblasts [3,4]. The functional effects of the dermis decrease as the skin ages due to several internal and external factors. Internal factors include hormone levels and genotypes, whereas external factors include lifestyle, chemical pollution, and ultraviolet (UV) radiation [5,6].

UV radiation is a major external factor causing photoaging of the skin; it induces DNA damage, denaturation of proteins, and production of reactive oxygen species (ROS) in the skin and triggers the degradation of the ECM [7]. ROS plays an important role in chemical breakdown, causing damage to biological structures, and food spoilage. Excessive ROS production can cause protein denaturation and lipid peroxidation. It also interferes with antioxidant defense mechanisms and induces inflammatory responses [8,9]. ROS generation increases collagenase matrix metalloproteinase (MMP-1, MMP-3, and MMP-9) synthesis and degrades collagen in the dermis [10]. Furthermore, UV radiation induces the inhibition of the transforming growth factor beta (TGF-β) signaling pathway that synthesizes collagen and fibronectin [11].

UV radiation stimulates pro-inflammatory factors, such as tumor necrosis factor-alpha (TNF-α), cyclooxygenase-2 (COX-2), and interleukin 1 (IL-1) [12]. TNF-α induces intercellular ROS production and several molecular pathways related to the aging process, such as mitogen-activated protein kinase (MAPKs), nuclear factor kappa B (NF-κB), and activator protein 1 (AP-1) [13,14]. MAPKs regulate the transcription factors NF-κB and AP-1, which promote MMP-1 expression [15]. During this process, skin aging progresses, and wrinkles, pigmentation, and sagging appear on the skin [16]. Consequently, the inhibition of ROS production and TNF-α is the key to preventing skin aging.

In a previous study, 2-*O*-*β*-d-glucopyranosyl-4,6-dihydroxybenzaldehyde (GDHBA) was isolated from maqui berry (*Aristotelia chilensis*) as a novel natural product, but the oxidative and inflammatory responses of this compound in human dermal fibroblasts (HDFs) damaged by UV radiation and TNF-α have not been elucidated [17,18,19]. In our previous study, we examined plant-derived agents that could prevent skin aging and found that hot water extract from *Morus alba* fruits significantly inhibited MMP-1 secretion in TNF-α-stimulated HDFs [20]. Furthermore, nine compounds, including oddioside A, from the 21 compounds isolated from the hot water extract significantly inhibited MMP-1 secretion [20]. Therefore, in the current study, we focused on these compounds. Among the compounds obtained from *M. alba* fruits, GDHBA exhibited an inhibitory effect on MMP-1 secretion and structural specificity. Therefore, the effects of GDHBA on oxidative and inflammatory responses in TNF-α-treated HDFs were evaluated in this study.

## 2. Results

### 2.1. Effect of GDHBA on ROS, Nitric Oxide (NO), and Prostaglandin-E2 (PGE_2_) Levels in TNF-α-Induced HDFs

**Figure 1:**
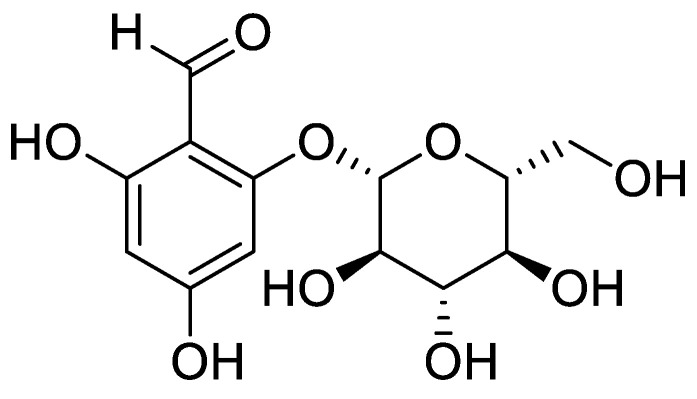
Structure of 2-*O*-*β*-d-glucopyranosyl-4,6-dihydroxybenzaldehyde (GDHBA).

In our previous study, GDHBA (Figure 1) isolated from *M. alba* fruits inhibited MMP-1 secretion in TNF-α-induced HDFs [20], suggesting that GDHBA may inhibit the oxidative and inflammatory responses that occur during intrinsic and extrinsic aging processes. Continuous exposure to UV radiation induces the production of pro-inflammatory cytokine TNF-α and ROS. Excessively increased TNF-α and ROS levels lead to several inflammatory responses and aging. Therefore, TNF-α and ROS production can be analyzed to investigate mechanisms similar to the processes involved in UV-mediated skin damage. Therefore, we examined the expression levels of ROS, NO, and PGE_2_ to investigate the possible suppression of GDHBA oxidation in HDFs.

First, HDFs were treated with TNF-α, which is both an oxidative stress stimulator and an inflammatory response inducer. The number of intracellular free radicals produced was measured to determine the level of oxidative stress. Dichlorofluorescein diacetate (DCFDA) was used as the probe. DCFDA, a non-fluorescent material, exhibits high fluorescence when de-esterified and oxidized in cells.

**Figure 2:**
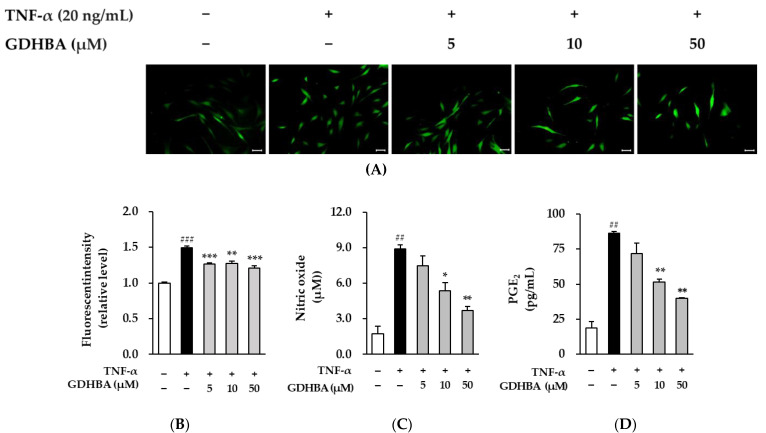
Effect of GDHBA on ROS (**A**,**B**), NO (**C**), and PGE_2_ production (**D**) in TNF-α stimulated HDFs. HDFs were pretreated with 5, 10, and 50 μM GDHBA for 1 h and incubated for 15 min and 24 h with TNF-α (20 ng/mL). Scale bar, 50 μm (**A**,**B**) Fluorescence intensity, and ROS production were observed using 10 µM DCFDA for 15 min. (**B**) Nitrite content in the cell culture supernatant was measured using the Griess assay. (**C**) PGE_2_ production was quantified using an ELISA kit. Each bar represents mean ± SEM. ## *p* < 0.01 and ### *p* < 0.001 versus normal control. * *p* < 0.05, ** *p* < 0.01, and *** *p* < 0.001 versus stimulus alone.

As shown in Figure 2A, the fluorescence intensity increased in TNF-α-treated HDFs compared to that in the normal control cells, whereas it decreased upon treatment with 5, 10, and 50 µM GDHBA. As shown in Figure 2B, ROS production increased by 1.49 ± 0.0-fold (*p* < 0.001) in TNF-α-induced HDFs compared to that in the normal control cells. Meanwhile, ROS production significantly decreased in the GDHBA treatment group compared to that in the TNF-α treatment group (5 µM: 15.4%, 10 µM: 14.8%, 50 µM: 18.8%). These results indicate that GDHBA has ROS-scavenging activity.

ROS-induced NO is an inflammatory mediator produced by nitric oxide synthase (NOS) and is associated with innate immunity [21]. We assessed the influence of GDHBA on TNF-α-induced NO levels. NO production increased in TNF-α-induced HDFs to 8.90 ± 0.33-fold (*p* < 0.01). NO production decreased in a concentration-dependent manner upon treatment with GDHBA compared to that in the TNF-α treatment group (5 µM: 15.7%, 10 µM: 39.9%, 50 µM: 58.4%) (Figure 2C).

Prostaglandins (PGs) are involved in the inflammatory response; among them, PGE2 is synthesized by COX-2 [22]. We examined the influence of GDHBA on TNF-α-induced PGE_2_ levels. TNF-α treatment increased the PGE_2_ level compared to that in the control group (86.4 ± 1.05 pg/mL, *p* < 0.01). PGE_2_ levels decreased in a concentration-dependent manner upon treatment with GDHBA compared to that of the TNF-α treatment group (5 µM: 16.7%, 10 µM: 40.5%, 50 µM; 53.8%) (Figure 2D).

### 2.2. Effect of GDHBA on MMP-1 and Type I Procollagen (COLIA1) Secretion in TNF-α-Induced HDFs

Collagen is broken down by the collagenase MMP-1; thus, MMP-1 inhibitors may decrease collagen breakdown during skin damage. Therefore, we investigated whether GDHBA could prevent skin aging in TNF-α-stimulated HDFs. MMP-1 secretion increased by 1.70 ± 0.02-fold (*p* < 0.001) compared to that in the normal control. GDHBA (50 μM) inhibited the TNF-α-induced increase in MMP-1 expression. MMP-1 secretion decreased upon treatment with GDHBA compared to that in the TNF-α stimulated group (5 µM: 34.1%, 10 µM: 42.4%, 50 µM: 55.3%). We evaluated the effect of GDHBA treatment on COLIA1 and observed that TNF-α treatment decreased its secretion (0.13 ± 0.00-fold, *p* < 0.001) compared to that of the normal control cells. Decreased COLIA1 concentration tended to increase by 207.7% after 50 μM GDHBA treatment compared to that of the TNF-α-stimulated group.

### 2.3. Effect of GDHBA on Phosphorylation of MAPKs in TNF-α-Induced HDFs

MAPK phosphorylated by UV radiation and TNF-α upregulates the expression of MMP-1 [15,23]; therefore, the influence of GDHBA on the phosphorylation of MAPKs was investigated. HDFs were pretreated with 5, 10, or 50 µM GDHBA for 1 h and stimulated with TNF for 15 min. Western blotting was performed to determine MAPK expression. The levels of phosphorylated forms of p38, ERK, and JNK increased in the TNF-α-treated group (p38: 3.12 ± 0.01-fold, *p* < 0.001; ERK: 1.88 ± 0.02-fold, *p* < 0.001; JNK: 1.66 ± 0.06-fold, *p* < 0.05). However, phosphorylation levels of p38, ERK, and JNK decreased by GDHBA treatment in a concentration-dependent manner. 50 µM GDHBA significantly suppressed the phosphorylation of all MAPKs compared to that in the TNF-α-stimulated group (p-p38: 35.9%, p-ERK: 47.9%, p-JNK: 49.4%). GDHBA (50 µM) suppressed p-p38 phosphorylation level by 35.9%. Moreover, p-ERK and p-JNK levels decreased in a concentration-dependent manner (p-ERK; 5 µM: 23.9%, 10 µM: 37.8%, 50 µM: 47.9%) (p-JNK; 5 µM: 20.5%, 10 µM: 38.6%, 50 µM: 49.4%).

### 2.4. Effect of GDHBA on Phosphorylation of c-Jun, NF-κB, and COX-2 in TNF-α Induced HDFs

TNF-α-induced excessive ROS expression increases the phosphorylation of c-Jun and NF-kB and the expression of COX-2 [24,25,26]. Therefore, the influence of GDHBA on the phosphorylation of c-Jun and NF-κB and the expression of COX-2 was investigated. HDFs were pretreated with 5, 10, and 50 µM GDHBA for 1 h and stimulated with TNF-α for 15 min and 6 h. MAPK expression was determined using Western blotting. The phosphorylated forms of c-Jun and NF-κB and the expression of COX-2 increased in the TNF treatment group (c-Jun: 1.40 ± 0.01-fold, *p* < 0.001; NF-κB: 10.01 ± 0.12-fold, *p* < 0.01; COX-2: 5.81 ± 0.03-fold, *p* < 0.001). However, phosphorylation of c-Jun, NF-κB and the expression of COX-2 decreased in the 50 µM GDHBA-treated group (p-c-Jun: 32.1%, p-NF-κB: 55.9%, COX-2: 31%). Moreover, p-c-Jun and p-NF-κB levels decreased by GDHBA in a concentration-dependent manner (p-c-Jun; 5 µM: 20.7%, 10 µM: 26.4%, 50 µM: 32.1%) (p-NF-κB; 5 µM: 21.9%, 10 µM: 23.9%, 50 µM: 55.9%) (COX-2; 5 µM: 39.9%, 10 µM: 30.8%, 50 µM: 31%).

## 3. Discussion

Humans experience intrinsic aging through hormonal changes or extrinsic aging through UV radiation, smoking, and chemicals. The skin is directly exposed to external environmental factors and undergoes aging [27]. UV radiation, one of the several causes of extrinsic aging, is responsible for skin damage. In recent years, the development of cosmetics and therapeutic agents to ameliorate these lesions of skin aging has focused on natural compounds that are free from harmful synthetic materials [28,29,30]. Therefore, we investigated the compounds isolated from *M. alba* fruits that inhibit collagenase expression.

Fibroblasts, which are mostly found in the dermal layer of the skin and produce ECM components, endure severe damage during the skin aging process [31]. In aged skin, the ability of fibroblasts to produce collagen type I is statistically reduced [32]. An important feature of aging skin is the destruction of fibroblasts, which overproduce collagenase MMP-1 and reduce the levels of ECM proteins, including collagen and elastin [33]. UV radiation-induced skin cell damage involves the production of collagenase MMP-1 via TNF-α and ROS production [34]. TNF-α and ROS activate the phosphorylation of p38, ERK, and JNK and the phosphorylation of two sub-proteins of AP-1, namely c-Fos and c-Jun. It also causes the phosphorylation and translocation of NF-κB [15,35]. MMPs degrade ECM proteins, including elastin and type I collagen [36].

In our previous study, the extract of *M. alba* fruit and the compounds isolated from the extract exhibited a protective effect against skin damage [15]. As a follow-up, in this study, we focused on the effects of GDHBA. GDHBA was first isolated from a maqui berry (*A. chilensis*); however, its biological and pharmacological effects have not been reported, except for its hydroxyl radical scavenging effect [17]. Therefore, this is the first report on the biological effects of GDHBA at the cellular level.

ROS are formed as a result of normal cellular activity and participate in cell signaling [37]. Cellular ROS levels can be divided into two groups. Certain processes release ROS through mitochondrial oxidative metabolism and induce ROS signaling pathways as a part of cellular defense mechanisms against bacterial invasion and cytokines [38]. ROS causes inflammation in both intrinsic and extrinsic aging, and inflammatory responses accelerate skin aging. Furthermore, ROS promotes the synthesis of MMPs and induces collagen degradation [39]. Production of intracellular ROS was investigated to measure the antioxidant capacity of GDHBA and it was found that GDHBA was effective in suppressing TNF-α-induced ROS increase (Figure 2A,B).

Inflammatory responses occur when inflammatory mediators, including TNF-α, NO, and PGE_2_, are stimulated. NO is an important regulatory and effector molecule with diverse biological functions [40]. Low concentrations of NO protect the cardiovascular system, whereas excessive NO expression promotes inflammation and oxidative stress [41,42]. NO production was investigated to measure the anti-inflammatory and anti-oxidative stress tolerance capacity of GDHBA, and we found that GDHBA was effective in suppressing TNF-α-induced increase in NO levels (Figure 2C).

PGs are hormones derived from arachidonic acid and are involved in inflammatory responses and contribute to tumorigenesis [43,44]. PG production increases in UV radiation-exposed skin, and the level of PGE_2_, one of the PGs, increases with aging and skin inflammation [45,46]. The expression of COX-2, an important factor involved in PGE_2_ synthesis, is upregulated by ROS and TNF-α [22]. We investigated PGE_2_ production to measure the anti-inflammatory response capacity of GDHBA treatment and found that GDHBA was effective in suppressing the TNF-α-induced increase in PGE_2_ levels (Figure 2D).

**Figure 3:**
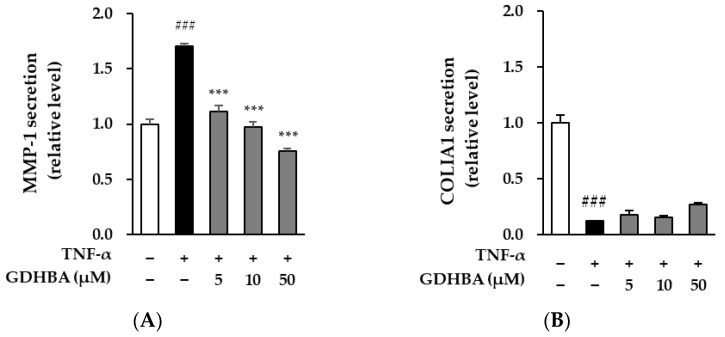
Effect of GDHBA on MMP-1 (**A**) and COLIA1 (**B**) secretion in TNF-α stimulated HDFs. HDFs were pretreated with 5, 10, and 50 μM of GDHBA. (**A**,**B**) for 1 h and then incubated for 24 h with TNF-α (20 ng/mL). MMP-1 and COLIA1 secretion was quantified using an ELISA kit. Each bar represents mean ± SEM. ### *p* < 0.001 versus normal control. *** *p* < 0.001 versus stimulus alone.

MMP-1 is an MMP enzyme that degrades various types of collagen, leading to connective tissue destruction and complete damage to the dermis. The presence of MMP-1 damages the skin dermis, resulting in loss of elasticity, formation of wrinkles, and sagging [47,48]. Conversely, procollagen, the precursor of collagen, promotes collagen protein synthesis and plays an important role in skin elasticity and structure [49]. Therefore, the inhibition of ECM degradation may have a protective effect against UV radiation-induced skin damage. The effect of GDHBA on TNF-α-induced HDFs was evaluated by measuring the changes in MMP-1 and COLIA1 levels, as they affect skin wrinkling. The results revealed that GDHBA decreased and restored the secretion of MMP-1 and COLIA1, respectively (Figure 3).

**Figure 4:**
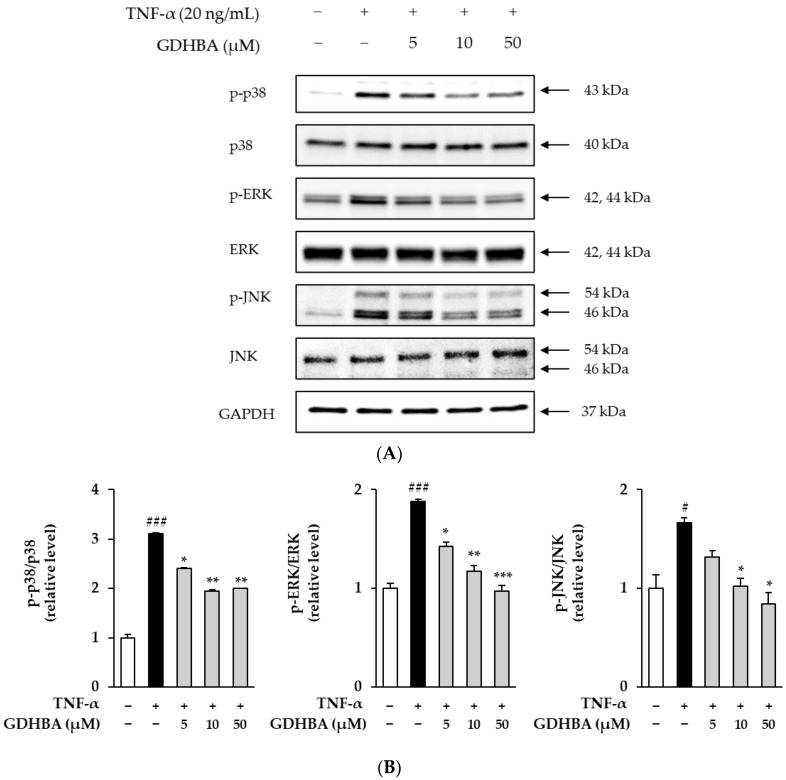
Effect of GDHBA on phosphorylation of MAPKs in TNF-α stimulated HDFs. HDFs were pretreated with 5, 10, and 50 μM of GDHBA for 1 h and then incubated with TNF-α (20 ng/mL) for 15 min. Expression of p-p38, p38, p-ERK, ERK, p-JNK, JNK, and GAPDH was determined using Western blotting. Each bar represents mean ± SEM. # *p* < 0.05 and ### *p* < 0.001 versus normal control. * *p* < 0.05, ** *p* < 0.01, and *** *p* < 0.001 versus stimulus alone.

According to several previous studies, MMP expression is a key factor in controlling skin damage caused by the NF-κB, AP-1, and MAPK signaling pathways [23,50,51]. NF-κB and AP-1 are phosphorylated in response to the phosphorylation of MAPKs, which are activated by the p38, ERK, and JNK signaling pathways. TNF-α treatment overinduced the phosphorylation of p38, ERK, and JNK, which was significantly inhibited by 50 μM GDHBA co-treatment (Figure 4). The NF-κB protein complex, triggered by UV radiation, ROS, and pro-inflammatory cytokines, is crucial for an immune response [28]. Moreover, activated NF-κB causes collagen degradation by promoting MMP development [52,53].

**Figure 5:**
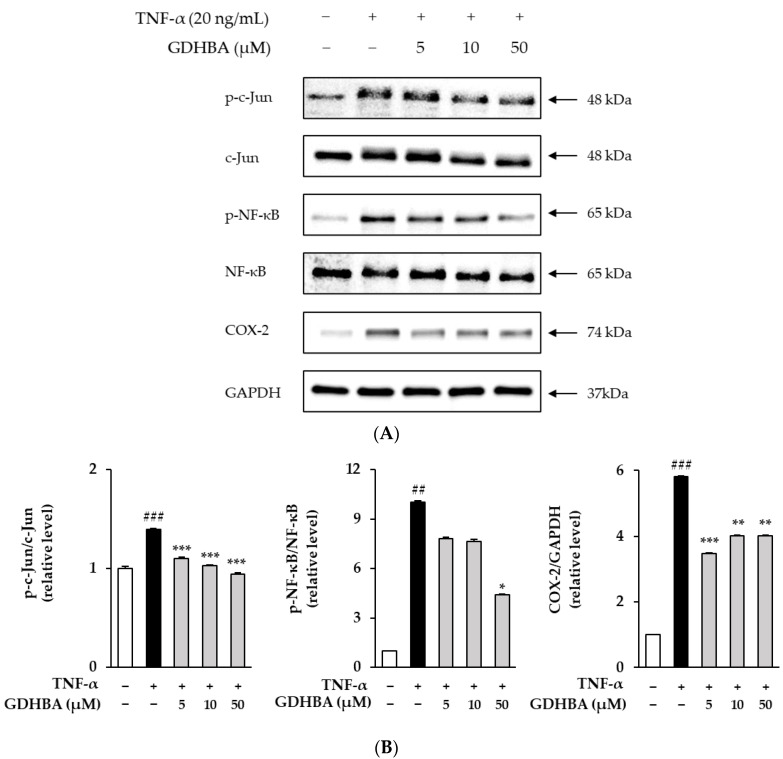
Effect of GDHBA on c-Jun, NF-κB, and COX-2 levels in TNF-α-treated HDFs. HDFs were pretreated with 5, 10, and 50 μM GDHBA (**A**,**B**) for 1 h and then incubated for 15 min and 6 h with TNF-α (20 ng/mL). Western blotting was performed to measure the expression of p-c-Jun, c-Jun, p-NF-κB NF-κB, COX-2, and GAPDH. Each bar represents mean ± SEM. ## *p* < 0.01 and ### *p* < 0.001 versus normal control. * *p* < 0.05, ** *p* < 0.01, and *** *p* < 0.001 versus stimulus alone.

COX-2 is a pro-inflammatory mediator that converts arachidonic acid into PGs such as PGE_2_, promoting skin aging and inflammatory skin diseases [54]. Inflammatory stimulation of healthy human fibroblasts activates COX-2, and PGE_2_ assists dendritic cell movement and promotes IL-23 expression [55]. We investigated whether GDHBA has anti-inflammatory effects and found that the levels of the phosphorylated forms of c-Jun, NF-κB, and COX-2 were significantly increased in TNF-α-stimulated HDFs and were significantly decreased by 50 μM GDHBA (Figure 5).

The skin ages due to direct exposure to external environmental factors, and one of the various external factors–UV radiation–is the main cause of skin damage. Skin damage causes oxidative stress owing to persistent UV radiation exposure, leading to an inflammatory response and wrinkle formation. Fibroblasts are mainly found in the dermal layer of the skin; they produce ECM components and are one of the most damaged cells in the process of skin aging. UV radiation-induced damage to skin cells induces pro-inflammatory cytokines, such as TNF-α, and ROS. They promote the expression of collagenases, such as MMP-1, causing wrinkles and reduced elasticity. MMPs play important roles in the process of skin damage and are regulated by the MAPK, NF-κB, and AP-1 signaling pathways. GDHBA had inhibitory and restorative effects on MMP-1 and procollagen type I in TNF-α-treated HDFs and decreased the activation of MAPKs and NF-κB. Therefore, GDHBA may effectively inhibit skin damage. Moreover, since GDHBA inhibits the expression of NO, PGE_2_, and COX-2, it could have an inhibitory effect against inflammatory reactions in HDFs.

## 4. Materials and Methods

### 4.1. Isolation of GDHBA

GDHBA was isolated from *M. alba* fruits (mulberry) as previously described [20]. Briefly, a hot water extract (1.0 kg) was obtained from fresh mulberry (5.0 kg), which was subjected to chromatography on a Diaion HP-20 column to obtain 21 fractions (Fr.1–Fr.21). Fr.8 was fractionated further by silica gel column chromatography to obtain GDHBA (4.6 mg).

### 4.2. Sample Preparation

GDHBA was dissolved in 10 mM dimethyl sulfoxide (DMSO; Sigma-Aldrich, St. Louis, MO, USA). TNF-α (PeproTech, Rocky Hill, NJ, USA) was dissolved in 1% bovine serum albumin fraction V (BSA; Merck, Darmstadt, Germany) solution and stored at −20 °C.

### 4.3. Cell Culture

HDFs were obtained from PromoCell GmbH (Heidelberg, Germany). The cells were cultured in DMEM containing 10% fetal bovine serum (FBS; Atlas, Fort Collins, CO, USA) and 1% antibiotics. Subculturing was performed till the cells reached 80–90% confluency in 100 mm dishes. Cell culture was performed in an incubator maintained at 5% CO_2_ and 37 °C.

### 4.4. Cell Viability

HDFs were plated in clear bottom 48-well cell culture plates (2 × 10^4^ cells/well) and incubated for 24 h; the culture medium was changed to serum-free medium (DMEM without FBS) and incubated for 24 h. Then, the cells were treated with a specific concentration (μM) of GDHBA and incubated for 24 h. To evaluate the cytotoxicity of GDHBA, the supernatant was discarded, 10% EZ-Cytox solution in serum-free medium (DoGenBio, Seoul, Republic of Korea; 100 μL per well) was added, and the plate was incubated for 1 h. Absorbance was measured using a SPARK 10M microplate reader (Tecan, Männedorf, Switzerland) at 450 nm.

### 4.5. Enzyme-Linked Immunosorbent Assay (ELISA)

HDFs were plated in clear bottom 48-well cell culture plates (2 × 10^4^ cells/well) and incubated for 24 h; the culture medium was changed to serum-free medium (DMEM without FBS) and incubated for 24 h. The cells were treated with 5, 10, and 50 μM GDHBA for 1 h before exposure to 20 ng/mL TNF-α for 24 h. Absorbance was measured using a SPARK 10M microplate reader at 450 nm.

### 4.6. ROS Assay

HDFs were plated in 96-well black plates (1 × 10^4^ cells/well) and incubated for 24 h; the culture medium was changed to serum-free medium (DMEM without FBS) and incubated for 24 h. Then, the cells were treated with 5, 10, and 50 μM GDHBA for 1 h before exposure to 20 ng/mL TNF-α and 10 μM DCFDA (Sigma-Aldrich) for 15 min. Fluorescence was measured using a SPARK 10M microplate reader at 485/535 nm.

### 4.7. NO Assay

HDFs were plated in 96-well black plates (1 × 10^4^ cells/well) and incubated for 24 h; the culture medium was changed to serum-free medium (DMEM without FBS) and incubated for 24 h. The cells were treated with 5, 10, and 50 μM GDHBA for 1 h before exposure to 20 ng/mL TNF-α for 24 h. Griess assay was performed to measure the concentration of nitrite in the supernatant. The supernatants were incubated with 1% sulfanilamide, 0.1% *N*-(1-naphthyl)-ethylenediamine, and 5% phosphoric acid for 10 min at room temperature. Nitrite content was measured using a SPARK 10M microplate reader at 540 nm. The level of NO produced in each sample was determined using a standard sodium nitrite (NaNO_2_) curve.

### 4.8. Western Blotting

HDFs were plated in clear bottom 6-well cell culture plates (3 × 10^5^ cells/well) and incubated for 24 h; the culture medium was changed to serum-free medium (DMEM without FBS) and incubated for 24 h. The cells were treated with 5, 10, and 50 μM GDHBA for 1 h before exposure to 20 ng/mL TNF-α for 15 min or 6 h. Cell samples for the investigation of MAPK, c-Jun, and NF-κB expression were exposed to TNF-α for 15 min, and the samples for the investigation of COX-2 expression were exposed to TNF-α for 6 h. After washing with DPBS, the cells were lysed using 1× radioimmunoprecipitation assay (RIPA) buffer (Tech & Innovation, Gangwon, Republic of Korea). The protein concentration was quantified using a BCA Protein Assay Kit (Thermo Scientific, Waltham, MA, USA). Equal amounts of protein were loaded onto polyacrylamide gels and separated by electrophoresis. The proteins were then transferred onto polyvinylidene difluoride (PVDF) membranes. The membrane was placed in TBS-Tween20 (TBS-T; Thermo Fisher Scientific, Waltham, MA, USA) containing 5% skim milk and allowed to react for 1 h. Primary and secondary antibodies were incubated overnight at 4 °C and room temperature, respectively, for 2 h.

Finally, protein bands were observed using Super Signal^®^ West Femto Maximum Sensitivity Chemiluminescent Substrate (Thermo Fisher Scientific) and a Fusion Solo Chemiluminescence System (PEQLAB Biotechnologie GmbH, Erlangen, Germany). Protein bands were quantified using the ImageJ program (TotalLab, Newcastle, UK).

### 4.9. Statistical Analyses

GraphPad Prism version 8.0.1 (GraphPad Software Inc., La Jolla, CA, USA), a statistical program, was used to analyze the experimental results. Data are expressed as mean ± standard error of the mean (SEM). After analysis by one-way ANOVA, statistical significance was confirmed by Tukey’s test at *p* < 0.05.

## 5. Conclusions

We investigated the effect of GDHBA, isolated from *M.* alba fruit, on damage to TNF-α-induced HDFs. GDHBA decreased oxidative stress-induced ROS levels in TNF-α-stimulated HDFs and decreased the expression of the inflammatory mediators NO and PGE_2_. It also inhibited MMP-1 expression and increased COLIA1 secretion. GDHBA suppressed the phosphorylation of p38, ERK, JNK, c-Jun, and NF-κB and the expression of COX-2. Thus, GDHBA suppressed the damage in HDFs by regulating oxidative and inflammatory responses under TNF-α-stimulated conditions. Although additional experiments are needed to understand the effects of GDHBA on skin damage, it is a potential agent that can ameliorate skin damage and prevent anti-inflammatory effects.

## Data Availability

Not applicable.

## Abbreviations

DMSO	Dimethyl Sulfoxide
ECM	Extracellular matrix
ELISA	Enzyme-linked immunosorbent assay
FBS	Fetal bovine serum
HDFs	Human dermal fibroblasts
MAPK	Mitogen-activated protein kinase
MMP-1	Matrix metalloproteinase-1
NOS	Nitric oxide synthase
PG	Prostaglandin
PVDF	Polyvinylidene difluoride
ROS	Reactive oxygen species
SEM	Standard error of mean
TNF-α	Tumor necrosis factor-alpha

## References

[1] Benson H.A., Watkinson A.C. (2012). Topical and Transdermal Drug Delivery: Principles and Practice.

[2] Uitto J. (1989). Connective tissue biochemistry of the aging dermis: Age-associated alterations in collagen and elastin. Clin. Geriatr. Med..

[3] Strnadova K., Sandera V., Dvorankova B., Kodet O., Duskova M., Smetana K., Lacina L. (2019). Skin aging: The dermal perspective. Clin. Dermatol..

[4] Tzaphlidou M. (2004). The role of collagen and elastin in aged skin: An image processing approach. Micron.

[5] Cao C., Xiao Z., Wu Y., Ge C. (2020). Diet and skin aging—From the perspective of food nutrition. Nutrients.

[6] Parrado C., Mercado-Saenz S., Perez-Davo A., Gilaberte Y., Gonzalez S., Juarranz A. (2019). Environmental stressors on skin aging. Mechanistic insights. Front. Pharmacol..

[7] Gilchrest B.A., Park H.-Y., Eller M.S., Yaar M. (1996). Mechanisms of ultraviolet light-induced pigmentation. Photochem. Photobiol..

[8] Hahn H.J., Kim K.B., An I.S., Ahn K.J., Han H.J. (2017). Protective effects of rosmarinic acid against hydrogen peroxide-induced cellular senescence and the inflammatory response in normal human dermal fibroblasts. Mol. Med. Rep..

[9] Das P., Ganguly S., Margel S., Gedanken A. (2021). Immobilization of heteroatom-doped carbon dots onto nonpolar plastics for antifogging, antioxidant, and food monitoring applications. Langmuir.

[10] Brenneisen P., Sies H., Scharffetter-Kochanek K. (2002). Ultraviolet-B irradiation and matrix metalloproteinases: From induction via signaling to initial events. Ann. N. Y. Acad. Sci..

[11] Rittié L., Fisher G.J. (2002). UV-light-induced signal cascades and skin aging. Ageing Res. Rev..

[12] Hasham R., Choi H.-K., Sarmidi M.R., Park C.-S. (2013). Protective effects of a Ficus deltoidea (*Mas cotek*) extract against UVB-induced photoageing in skin cells. Biotechnol. Bioprocess Eng..

[13] Wang T., Zhang X., Li J.J. (2002). The role of NF-κB in the regulation of cell stress responses. Int. Immunopharmacol..

[14] Bickers D.R., Athar M. (2006). Oxidative stress in the pathogenesis of skin disease. J. Investig. Dermatol..

[15] Phung H.M., Lee S., Hong S., Lee S., Jung K., Kang K.S. (2021). Protective Effect of Polymethoxyflavones Isolated from Kaempferia parviflora against TNF-α-Induced Human Dermal Fibroblast Damage. Antioxidants.

[16] Lee D.H., Oh J.-H., Chung J.H. (2016). Glycosaminoglycan and proteoglycan in skin aging. J. Dermatol. Sci..

[17] Li J., Yuan C., Pan L., Benatrehina P.A., Chai H., Keller W.J., Naman C.B., Kinghorn A.D. (2017). Bioassay-guided isolation of antioxidant and cytoprotective constituents from a maqui berry (*Aristotelia chilensis*) dietary supplement ingredient as markers for qualitative and quantitative analysis. J. Agric. Food Chem..

[18] Benatrehina P.A., Pan L., Naman C.B., Li J., Kinghorn A.D. (2018). Usage, biological activity, and safety of selected botanical dietary supplements consumed in the United States. J. Tradit. Complement. Med..

[19] Schmeda-Hirschmann G., Jiménez-Aspee F., Theoduloz C., Ladio A. (2019). Patagonian berries as native food and medicine. J. Ethnopharmacol..

[20] Kim K.S., Kim R., Son S.-R., Kang K.S., Jang D.S., Lee S. (2022). Oddioside A, a New Phenolic Glycoside Isolated from the Fruits of Morus alba (Mulberry), Protects TNF-α;-Induced Human Dermal Fibroblast Damage. Antioxidants.

[21] Richard S.A. (2021). Exploring the pivotal immunomodulatory and anti-inflammatory potentials of glycyrrhizic and glycyrrhetinic acids. Mediators Inflamm..

[22] Masaki H. (2010). Role of antioxidants in the skin: Anti-aging effects. J. Dermatol. Sci..

[23] Hwang E., Lin P., Ngo H.T., Gao W., Wang Y.-S., Yu H.-S., Yi T.-H. (2018). Icariin and icaritin recover UVB-induced photoaging by stimulating Nrf2/ARE and reducing AP-1 and NF-κB signaling pathways: A comparative study on UVB-irradiated human keratinocytes. Photochem. Photobiol. Sci..

[24] Lingappan K. (2018). NF-κB in oxidative stress. Curr. Opin. Toxicol..

[25] Onodera Y., Teramura T., Takehara T., Shigi K., Fukuda K. (2015). Reactive oxygen species induce Cox-2 expression via TAK1 activation in synovial fibroblast cells. FEBS Openbio.

[26] Liu Y., Wang Z., Xie W., Gu Z., Xu Q., Su L. (2017). Oxidative stress regulates mitogen-activated protein kinases and c-Jun activation involved in heat stress and lipopolysaccharide-induced intestinal epithelial cell apoptosis. Mol. Med. Rep..

[27] Huertas A.C.M., Schmelzer C.E., Hoehenwarter W., Heyroth F., Heinz A. (2016). Molecular-level insights into aging processes of skin elastin. Biochimie.

[28] Wang L., Lee W., Oh J.Y., Cui Y.R., Ryu B., Jeon Y.-J. (2018). Protective effect of sulfated polysaccharides from celluclast-assisted extract of Hizikia fusiforme against ultraviolet B-Induced skin damage by regulating NF-κB, AP-1, and MAPKs signaling pathways in vitro in human dermal fibroblasts. Mar. Drugs.

[29] Kerdudo A., Burger P., Merck F., Dingas A., Rolland Y., Michel T., Fernandez X. (2016). Development of a natural ingredient–Natural preservative: A case study. C. R. Chim..

[30] Kashif M., Akhtar N., Mustafa R. (2017). An overview of dermatological and cosmeceutical benefits of Diospyros kaki and its phytoconstituents. Rev. Bras. Farmacogn..

[31] Wang X., Hong H., Wu J. (2019). Hen collagen hydrolysate alleviates UVA-induced damage in human dermal fibroblasts. J. Funct. Foods..

[32] Varani J., Dame M.K., Rittie L., Fligiel S.E., Kang S., Fisher G.J., Voorhees J.J. (2006). Decreased collagen production in chronologically aged skin: Roles of age-dependent alteration in fibroblast function and defective mechanical stimulation. Am. J. Clin. Pathol..

[33] Fisher G.J., Varani J., Voorhees J.J. (2008). Looking older: Fibroblast collapse and therapeutic implications. Arch. Dermatol..

[34] Marcos-Garcés V., Molina Aguilar P., Bea Serrano C., García Bustos V., Benavent Seguí J., Ferrández Izquierdo A., Ruiz-Saurí A. (2014). Age-related dermal collagen changes during development, maturation and ageing—A morphometric and comparative study. J. Anat..

[35] Hwang E., Gao W., Xiao Y.k., Ngo H.T., Yi T.H. (2019). Helianthus annuus L. flower prevents UVB-induced photodamage in human dermal fibroblasts by regulating the MAPK/AP-1, NFAT, and Nrf2 signaling pathways. J. Cell. Biochem..

[36] Binic I., Lazarevic V., Ljubenovic M., Mojsa J., Sokolovic D. (2013). Skin ageing: Natural weapons and strategies. Evid. Based Complement. Altern. Med..

[37] Snezhkina A.V., Kudryavtseva A.V., Kardymon O.L., Savvateeva M.V., Melnikova N.V., Krasnov G.S., Dmitriev A.A. (2019). ROS generation and antioxidant defense systems in normal and malignant cells. Oxid. Med. Cell. Longev..

[38] Zhang J., Wang X., Vikash V., Ye Q., Wu D., Liu Y., Dong W. (2016). ROS and ROS-mediated cellular signaling. Oxid. Med. Cell. Longev..

[39] Fuller B. (2019). Role of PGE-2 and other inflammatory mediators in skin aging and their inhibition by topical natural anti-inflammatories. Cosmetics.

[40] Pierini D., Bryan N.S. (2015). Nitric oxide availability as a marker of oxidative stress. Advanced Protocols in Oxidative Stress III.

[41] Kingwell B.A. (2000). Nitric oxide-mediated metabolic regulation during exercise: Effects of training in health and cardiovascular disease. FASEB J..

[42] Levick S.P., Widiapradja A. (2020). The diabetic cardiac fibroblast: Mechanisms underlying phenotype and function. Int. J. Mol. Sci..

[43] Bishop-Bailey D., Calatayud S., Warner T.D., Hla T., Mitchell J.A. (2002). Prostaglandins and the regulation of tumor growth. J. Environ. Pathol. Toxicol. Oncol..

[44] Kabashima K., Nagamachi M., Honda T., Nishigori C., Miyachi Y., Tokura Y., Narumiya S. (2007). Prostaglandin E2 is required for ultraviolet B-induced skin inflammation via EP2 and EP4 receptors. Lab. Investig..

[45] Li Y., Lei D., Swindell W.R., Xia W., Weng S., Fu J., Worthen C.A., Okubo T., Johnston A., Gudjonsson J.E. (2015). Age-associated increase in skin fibroblast–derived prostaglandin E2 contributes to reduced collagen levels in elderly human skin. J. Investig. Dermatol..

[46] Shim J.H. (2019). Prostaglandin E2 induces skin aging via E-prostanoid 1 in normal human dermal fibroblasts. Int. J. Mol. Sci..

[47] Xia W., Hammerberg C., Li Y., He T., Quan T., Voorhees J.J., Fisher G.J. (2013). Expression of catalytically active matrix metalloproteinase-1 in dermal fibroblasts induces collagen fragmentation and functional alterations that resemble aged human skin. Aging Cell.

[48] Freitas-Rodriguez S., Folgueras A.R., Lopez-Otin C. (2017). The role of matrix metalloproteinases in aging: Tissue remodeling and beyond. Biochim. Biophys. Acta Mol. Cell Res..

[49] Heo S.-J., Jeon Y.-J. (2009). Protective effect of fucoxanthin isolated from Sargassum siliquastrum on UV-B induced cell damage. J. Photochem. Photobiol. B Biol..

[50] Rabe J.H., Mamelak A.J., McElgunn P.J., Morison W.L., Sauder D.N. (2006). Photoaging: Mechanisms and repair. J. Am. Acad. Dermatol..

[51] Pittayapruek P., Meephansan J., Prapapan O., Komine M., Ohtsuki M. (2016). Role of matrix metalloproteinases in photoaging and photocarcinogenesis. Int. J. Mol. Sci..

[52] Makarov S.S. (2000). NF-κB as a therapeutic target in chronic inflammation: Recent advances. Mol. Med. Today.

[53] Hwang B.-M., Noh E.-M., Kim J.-S., Kim J.-M., Hwang J.-K., Kim H.-K., Kang J.-S., Kim D.-S., Chae H.-J., You Y.-O. (2013). Decursin inhibits UVB-induced MMP expression in human dermal fibroblasts via regulation of nuclear factor-κB. Int. J. Mol. Med..

[54] Yang G., Im H.-J., Wang J.H.-C. (2005). Repetitive mechanical stretching modulates IL-1β induced COX-2, MMP-1 expression, and PGE2 production in human patellar tendon fibroblasts. Gene.

[55] Arasa J., Terencio M.C., Andrés R.M., Marín-Castejón A., Valcuende-Cavero F., Payá M., Montesinos M.C. (2019). Defective induction of COX-2 expression by psoriatic fibroblasts promotes pro-inflammatory activation of macrophages. Front. Immunol..

